# Einsam(er) seit der Coronapandemie: Wer ist besonders betroffen? – psychologische Befunde aus Deutschland

**DOI:** 10.1007/s11553-021-00837-w

**Published:** 2021-03-11

**Authors:** Sonia Lippke, Franziska Keller, Christina Derksen, Lukas Kötting, Tiara Ratz, Lena Fleig

**Affiliations:** 1 Department of Psychology & Methods, Jacobs University Bremen, Bremen, Deutschland; 2 MSB Medical School Berlin/Hochschule für Gesundheit und Medizin, Berlin, Deutschland

**Keywords:** Einsamkeit, COVID-19, Ängste, Bewältigungsstrategien, Kommunikation, Loneliness, Corona pandemic, Fears, Coping strategies, Communication

## Abstract

**Hintergrund:**

Soziale Isolation ist ein Risikofaktor für Einsamkeit und damit für gesundheitliche Beeinträchtigungen. Veränderungen im Zusammenhang mit der Coronapandemie in Deutschland gilt es besser zu verstehen.

**Fragestellung:**

Ziel der Studie war es, Einsamkeit und assoziierte Faktoren vor und während der Coronapandemie in Deutschland systematisch zu untersuchen. Die Fragestellungen waren: 1. Wie *einsam* fühlen sich Menschen vor und während der Coronapandemie? 2. Wie viele Menschen fühlen sich seit Beginn der Coronapandemie *einsamer*? 3. Wie viele Menschen berichten über *gesundheitliche Belastungen *während der Coronapandemie?

**Material und Methoden:**

Im Jahr 2019 (vor der Coronapandemie) wurden 1003 und im Jahr 2020 (während der Coronapandemie) 1050 Erwachsene online befragt (51 % Frauen; 18–90 Jahre).

**Ergebnisse:**

Es fühlten sich 10,8 % vs. 26,6 % der Befragten vor bzw. seit der Coronapandemie mehrfach pro Woche oder täglich einsam. Alleinlebende, Frauen und Jüngere fühlten sich häufiger einsam. Seit der Coronapandemie fühlten sich 30,8 % einsamer, v. a. Jüngere. Von starken gesundheitlichen Belastungen berichteten 18,9 %, dies hing mit jüngerem Alter, verschiedenen Sorgen/Ängsten und Einsamkeit zusammen.

**Diskussion:**

Die höhere Ausprägung der Einsamkeit und Sorgen während der Coronapandemie sollte bei verhaltensbezogenen Maßnahmen zur Prävention der psychischen und körperlichen Beeinträchtigungen sowie behördlichen Maßnahmen berücksichtigt werden. Jüngere Menschen und Alleinlebende könnten profitieren, indem sie zu gezielten Bewältigungsstrategien (z. B. angemessener Nutzung digitaler Medien) ermutigt werden.

## Abstract (en)

**Background:**

Social isolation is a risk factor for loneliness and hence for impaired health. It has not yet been extensively investigated which psychological changes the corona pandemic has brought about in Germany.

**Objectives:**

The aim was to systematically investigate loneliness before and during the corona pandemic in Germany. The research questions were as follows: (1) How lonely do people feel before and during the corona pandemic? (2) How many people feel lonelier since the beginning of the corona pandemic? (3) How many people are affected in their subjective health due to limitations following the corona pandemic?

**Materials and methods:**

In 2019 (before the corona pandemic) 1003 adults were interviewed and in 2020 (during the corona pandemic) 1050 adults were assessed. Both surveys were conducted online and were representative for Germany (51% women; age 18–90 years).

**Results:**

In all, 10.8% vs. 26.6% of Germans felt lonely on two or more days per week before the corona pandemic vs. during the pandemic. Singles, women and younger respondents felt lonely more often in both surveys. Since the corona pandemic, 30.8% felt lonelier, especially younger individuals. Furthermore, 18.9% reported severely impaired health. Feeling more affected was related to younger age, various fears and more frequent perception of loneliness.

**Conclusion:**

Increasing loneliness and social isolation as well as various fears should be considered in further infection control measures. In general, overcoming loneliness in younger individuals and those living alone should be supported by building adequate coping skills that help to reduce impairments and fears.

## Einleitung

Soziale Bindungen tragen zur körperlichen und psychischen Gesundheit bei, insbesondere in stressreichen Situationen [19]. Während der Coronapandemie erscheinen sie als besonders wichtige Ressource: Die behördlichen sowie individuellen Maßnahmen gegen die Ausbreitung der Coronaviruserkrankung („coronavirus disease 2019“, COVID-19) wie z. B. Quarantäne, Einhalten des Mindestabstands sowie Arbeiten von Zuhause haben dazu geführt, dass sich bei vielen Personen die Zahl sozialer Interaktionen reduziert hat und sie mehr Zeit alleine oder in der Kernfamilie verbringen [1, 4, 15].

Alleinsein kann positiv wirken, da Kreativität, Zielorientierung und Konzentration gefördert werden – was als Sinnfindung verstanden wird [12, 16], aber auch mit sozialer Isolation und Einsamkeit einhergehen kann. Während Einsamkeit ein subjektives Gefühl darstellt, lässt sich soziale Isolation objektiv erfassen [1]: Beispielsweise gab es in den letzten Jahren mehr Haushalte, in denen nur eine Person wohnte. Sowohl soziale Isolation als auch wahrgenommene Einsamkeit können die Gesundheit beeinträchtigen, z. B. stehen sie im direkten Zusammenhang mit einer erhöhten depressiven Symptomatik. Einsamkeit hängt mit erhöhtem Blutdruck, ungünstigerem Schlafverhalten und einem schwächeren Immunsystem zusammen [12, 16]. Auch steht der Beziehungsstatus bzw. die Wohnform mit wahrgenommener Einsamkeit im Zusammenhang (z. B. [22]). So berichten Alleinstehende und Alleinlebende häufiger, sich einsam zu fühlen, als Menschen in einer Partnerschaft. Daneben erhöht ein niedriger sozioökonomischer Status das Risiko für Einsamkeit [7, 11]. Auch wenn objektive soziale Isolation (bspw. durch Partnerlosigkeit oder Leben in einem Singlehaushalt) mit dem Gefühl der Einsamkeit einhergehen kann, hängt Einsamkeit stärker mit der Qualität als mit der Quantität der sozialen Interaktionen zusammen. Nach Cacioppo und Hawkley [3] ist dies auch darauf zurückzuführen, dass Einsamkeit mit bisherigen Erfahrungen, kulturellen Normen, sozialen Bedürfnissen, körperlichen Beeinträchtigungen und Diskrepanzen zwischen tatsächlichen und gewünschten Beziehungen zusammenhängt: Personen, die sich einsam fühlen, neigen dazu, sich in der Kommunikation zurückzuhalten oder ganz aus Interaktionen zurückzuziehen. Dies kann dazu führen, dass Personen ihre Umwelt negativer wahrnehmen und sich noch einsamer fühlen [3].

Das Einsamkeitsempfinden der in Deutschland lebenden Menschen war im März 2020 höher als in 2017 [1]. Dies könnte mit den gesellschaftlichen Einschränkungen im Rahmen der Coronapandemie zusammenhängen [1, 6, 15]. Wenn soziale Isolation und Einsamkeit ansteigen, dann sollte dies ernst genommen werden, da es auch mit einem erhöhten Risiko zusammenhängen kann, an einer Depression zu erkranken oder eine Angstsymptomatik zu entwickeln [3]. Dabei ist weniger die Intensität entscheidend, sondern die Dauer der sozialen Isolation und der empfundenen Einsamkeit, wie in einem Überblicksartikel zu Kindern, Jugendlichen und jungen Erwachsenen festgestellt wurde [14]. Um den möglichen negativen Konsequenzen der sozialen Isolation präventiv zu begegnen, ist es wichtig, auch in Zeiten der Coronapandemie qualitativ wertvolle und zufriedenstellende Beziehungen aufrechtzuerhalten und adäquate Bewältigungsstrategien auszuüben [9]. Soziale Isolation hängt jedoch nicht nur mit Einschränkungen der psychischen Gesundheit zusammen, sondern auch mit einer Zunahme von finanziellen Sorgen sowie damit, dass sich Personen weniger gesundheitsförderlich verhalten [3, 9, 10]. All dies ist relevant für Prävention und Gesundheitsförderung.

Um die bisherigen Zusammenhänge der Coronapandemie weiterführend zu untersuchen, werden in dieser Studie folgende Forschungsfragen mithilfe zweier unabhängiger repräsentativer Online-Surveys mit Erwachsenen untersucht: 1. a) Wie einsam fühlen sich die Menschen vor und während der Coronapandemie? b) Fühlen sich Alleinlebende vor und während der Coronapandemie einsamer als diejenigen, die mit anderen Menschen zusammenwohnen? c) Gibt es Unterschiede zwischen Frauen und Männern sowie d) hinsichtlich des Alters? 2. a) Wie viele Menschen fühlen sich seit Beginn der Coronapandemie einsamer und wie sind die Zusammenhänge mit Geschlecht, Wohnsituation sowie Alter? 3. Wie viele Menschen berichten über gesundheitliche Belastungen während der Coronapandemie und welche Rolle spielen a) Alter, Geschlecht, b) Einsamkeit, c) Sorgen/Ängste sowie d) Bewältigungsstrategien inklusive Kommunikation und Sinnfindung?

## Methoden

### Erhebung im Jahr 2019

Die Daten vor der Coronapandemie wurden im Rahmen der Weleda-Trendforschung 2019 erhoben, indem eine Befragung einer bevölkerungsrepräsentativen Stichprobe von 1003 Erwachsenen durch das Institut forsa erfolgte. Der Erhebungszeitraum erstreckte sich vom 15.07.2019 bis zum 19.07.2019. In der Online-Befragung wurden u. a. die Themenfelder Wohnsituation und soziales Umfeld sowie Gefühle von Einsamkeit und soziodemografische Charakteristika erhoben. In dieser Studie wurde neben soziodemographischen Variablen (Geschlecht, Alter und Wohnsituation) nur das Konstrukt *Einsamkeit *betrachtet. Dieses wurde erhoben mit dem Item „Wie häufig haben Sie sich einsam gefühlt“ aus der Center for Epidemiological Studies-Depression-(CES‑D)-Skala und den Antwortalternativen (angelehnt an Kohlmann & Gerbershagen^1^): „täglich“, „mehrfach pro Woche“, „einmal die Woche“, „selten“ und „nie“ [8]. Eine Änderung der Originalantwortalternativen aus der CES-D-Skala wurde entsprechend nach den Pilotierungen vorgenommen, denn aufgrund von wiederholten Rückmeldungen interessierter Bürger*innen bei öffentlichen Veranstaltungen zu Messmethoden wurde Überarbeitungsbedarf identifiziert [21]. Entsprechend wurde eine Gruppendiskussion durchgeführt, bei der die Originalformate verschiedener Fragebogen und die geänderte Version wie beschrieben getestet und diskutiert wurden. Die Teilnehmer*innen der Gruppendiskussion sprachen sich mehrheitlich für das geänderte Format aus: Zum einen empfanden viele Befragte die verbalen Anker „manchmal/gelegentlich“, „öfters/häufiger“ und „meistens/ständig“ als wertend. Stattdessen wünschten sie sich numerische Anker. Zum anderen war es einigen Befragten wichtig, zwischen „täglich“ (anstatt „meistens/ständig“) und „mehrfach pro Woche“ (anstatt „öfters/häufiger“) sowie zwischen „selten“ und „nie“ zu unterscheiden. Eine Umrechnung in Tage pro Woche erfolgte in Anlehnung an Cacioppo, Fowler and Christakis (2009) wie folgt: „selten“ und „nie“ wurde als 0,5 Tage rekodiert (bei den Autoren als 0–1 Tage gemessen); „einmal die Woche“ als 1,5 Tage (bei den Autoren als 1–2 Tage), „mehrfach pro Woche“ als 3,5 Tage (bei den Autoren als 3–4 Tage) und „täglich“ als 6 Tage (bei den Autoren als 5–7 Tage).

### Erhebung im Jahr 2020

Die Daten während der Coronapandemie wurden im Rahmen der Weleda-Trendforschung 2020 erhoben, indem gleichermaßen eine Befragung einer bevölkerungsrepräsentativen Stichprobe von 1050 Erwachsenen durch das Institut Bilendi erfolgte. Der Erhebungszeitraum erstreckte sich vom 08.06.2020 bis zum 15.06.2020. Ebenfalls in einer Online-Befragung wurden neben den Themenfeldern der Trendforschung 2019 (s. oben) Sorgen/Ängste, Bewältigungsstrategien inklusive Kommunikation und Sinnfindung sowie Beschwerden in der subjektiven Gesundheit im Zusammenhang mit den behördlichen und individuellen Einschränkungen im Kontext der Coronapandemie erhoben. Die konkreten Fragen und Antwortalternativen orientierten sich an anderen Erhebungen (z. B. [1, 6, 21, 22]). Zusätzlich zu der Frage nach der *Häufigkeit der Einsamkeit* wurde auch die *Veränderung der wahrgenommenen Einsamkeit* erhoben mit der Frage „Fühlen Sie sich jetzt einsamer als vor den Einschränkungen?“ und den Antwortalternativen „trifft überhaupt nicht zu“ und „trifft eher nicht zu“ (zusammengefasst zu „keine Zunahme der Einsamkeit“) sowie „trifft eher zu“ und „trifft voll und ganz zu“ (zusammengefasst zu „Zunahme der Einsamkeit“).

Die *Risikowahrnehmung* in Form der subjektiven Zugehörigkeit zur Risikogruppe für eine COVID-19-Erkrankung oder einen schweren Verlauf wurde erhoben, indem gefragt wurde „Gehören Sie (z. B. aufgrund Ihres Alters oder Vorerkrankungen) zur Coronarisikogruppe?“.

*Sorgen/Ängste* wurden mit der Frage „Was hat Sie seit Beginn der Coronakrise beunruhigt oder geängstigt?“ erfasst. Dafür sollten die folgenden Bereiche mit „überhaupt nicht“, „eher schwach“, „eher stark“ oder „sehr stark“ bewertet werden: a) Angst um Familie und Freund*innen; b) Angst, mich mit dem Coronavirus anzustecken; c) Angst, die hohen täglichen Anforderungen (z. B. Homeoffice, Kinderbetreuung, Pflege von Angehörigen) nicht bewältigen zu können; d) Angst, nicht mehr selbstbestimmt handeln zu können/in meiner Freiheit eingeschränkt zu werden; e) Angst vor finanziellen Folgen (Gehaltseinbußen, Kurzarbeit, Jobverlust); f) Angst vor der Zukunft unserer Gesellschaft; g) Angst, meine Tagesstruktur zu verlieren; h) Ich war durch die Nachrichten (in Zeitung, Fernsehen, Radio, Internet) beunruhigt; i) Angst, länger allein zu sein, meine sozialen Kontakte zu vernachlässigen/vor sozialer Isolation; j) Angst, mich in Angst zu verlieren; k) Angst, vor die Tür zu gehen/das Haus zu verlassen; l) Angst vor Konflikten mit meiner Familie; m) Angst vor Gewalt in meiner Familie; n) Angst, meinen Hausarzt, einen Facharzt, ein Krankenhaus aufzusuchen; o) Angst, meine Angehörigen in einem Krankenhaus oder Pflegeheim nicht mehr wiederzusehen; oder p) Angst, dass ein Angehöriger verstirbt und ich mich nicht verabschieden kann.

*Bewältigungsstrategien mittels Kommunikation *wurde operationalisiert durch die Frage „Wie haben Sie bisher versucht, die eingeschränkten persönlichen sozialen Kontakte auszugleichen?“ mit Antwortmöglichkeiten wie bei Veränderung der Einsamkeit (s. oben). Die einzuschätzenden Strategien waren: a) Ich habe mehr telefoniert. b) Ich war häufiger in privaten Videocalls. c) Ich war verstärkt in den sozialen Netzwerken (z. B. Facebook, Instagram) unterwegs. d) Ich habe mehr Text‑/Sprachnachrichten versendet. e) Ich habe mehr Fotos gepostet/geschickt. f) Ich habe häufiger aus dem Fenster geschaut. g) Ich habe mehr Serien geschaut. h) Ich habe nichts verändert.

*Bewältigungsstrategien mittels Sinnfindung und Beziehungsqualität* wurden gemessen durch die Frage „Wie steht es um die Beziehung zu Ihrem engsten sozialen Umfeld? (engstes Umfeld sind bspw. die Familie oder Freund*innen, mit denen wir zusammenleben, oder die uns am nächsten stehen und mit denen wir vieles teilen)“ mit Antwortmöglichkeiten wie bei Veränderung der Einsamkeit (s. oben). Die Bereiche waren a) Wir sind enger zusammengewachsen, geben uns Halt. b) Ich habe meine Familie/mein soziales Umfeld mehr zu schätzen gelernt. c) Soziale Konflikte durch zu viel Nähe haben zugenommen (z. B. Streit, rauer Ton, Unruhe). d) Wir reden mehr miteinander. e) Wir haben uns voneinander distanziert. f) Ich habe mir viele Sorgen um meine Lieben/meine Freund*innen gemacht.

Schließlich wurden *gesundheitliche Belastungen während der Coronapandemie *durch die Frage „Wie stark haben Sie unter den Einschränkungen während der Coronapandemie gelitten (psychisch und/oder physisch)?“ erhoben. Die Antwortalternativen waren „Gar nicht, es ging mir gut“, „Etwas, es ging mir manchmal nicht so gut“, „Relativ stark, die Situation hat mich zunehmend belastet“ oder „Sehr stark, es ging mir sehr schlecht“.

### Studienpopulation

An der Erhebung 2019 nahmen insgesamt 518 (51,5 %) Frauen und 485 (48,5 %) Männer im Alter von 18 bis 88 Jahren (*M* *=* 53,70; *SD* *=* 16,80) teil. 23,7 % hatten einen Hauptschulabschluss, 37,2 % einen mittleren Abschluss und 34,3 % Abitur oder einen Hochschulabschluss.

An der Erhebung 2020 nahmen insgesamt 535 (51,0 %) Frauen und 515 (49,0 %) Männer im Alter von 18 bis 90 Jahren (*M* *=* 49,88; *SD* *=* 17,33) teil. 54 % der Befragten gaben an, Kinder zu haben. Zudem berichteten 27 % alleine zu leben, 42 % lebten mit einer weiteren Person in einem Haushalt und 31 % der Befragten mit 2–16 weiteren Personen in einem Haushalt. Von den Befragten waren 60 % berufstätig. Zur Risikogruppe im Hinblick auf eine Erkrankung und einen schweren Verlauf von COVID-19 zählten sich 49,7 % der Befragten (*M* *=* 60,22 Jahre; *SD* *=* 14,21; insbesondere 60- bis 69-Jährige zählten sich mit 85 % zur Risikogruppe sowie ab 70-Jährige mit 93 %). Diejenigen, die sich nicht zur Coronarisikogruppe zählten, waren im Mittel jünger mit *M* *=* 39,66 Jahren (*SD* *=* 13,69). 33 % der Befragten gaben an, während der Coronapandemie im Homeoffice gearbeitet zu haben. Von denjenigen, die im Homeoffice gearbeitet haben, gaben 84 % an, Kinder im betreuungspflichtigen Alter (<1 Jahr bis 14 Jahre) zu haben. Ebenso gehörten 24 % der Homeoffice-Tätigen zur „Rush hour of life“(RHOLer)-Gruppe. Diese Gruppe von 30- bis 39-Jährigen wird entsprechend bezeichnet, da ihr Anteil von Arbeit (bezahlt und unbezahlt) typischerweise höher ist als ihr Freizeitanteil [25]. Bei beiden Erhebungen wurde entsprechend der ethischen Standards der 1964 Helsinki-Deklaration, sowie der Guten Wissenschaftlichen Praxis der Deutschen Forschungsgemeinschaft (DFG) eine informierte Einwilligung eingeholt sowie alle Daten entsprechend erhoben und gespeichert.

### Statistische Analysen

Neben Faktoren- und Reliabilitätsanalysen zur Bildung von Skalen in Bezug auf Ängste und Bewältigungsstrategien wurden Frequenzanalysen, Pearson-Korrelationen, Varianzanalysen (Forschungsfragen 1–2) und eine lineare Regressionsanalyse (Forschungsfrage 3) mit IBM SPSS Statistics Version 26 berechnet.

## Ergebnisse

### Einsamkeit vor und während der Coronapandemie (Forschungsfrage 1)

Die berichtete *Einsamkeit* von Frauen und Männern sowie verschiedener Altersgruppen ist Tab. 1 zu entnehmen. Der Anteil derjenigen, die sich mehrfach pro Woche oder täglich einsam gefühlt haben, war mit 10,8 % vor der Coronapandemie im Jahr 2019 niedriger als während der Coronapandemie im Jahr 2020 mit 26,6 %. In beiden Stichproben waren jüngere Personen eher dazu geneigt, von häufigen Einsamkeitsgefühlen zu berichten, als ältere Befragte (Tab. 1).

**Tab. 1:** Gefühlte Einsamkeit der befragten Personen im Erhebungszeitraum 2019 und 2020

		Häufigkeit der Einsamkeit (*n *[%])				
		Selten/nie (0,5 Tage)	Einmal die Woche (1,5 Tage)	Mehrfach pro Woche (3,5 Tage)	Täglich (6 Tage)	Kennwert bzw. Teststatistik
2019	Gesamt (*n* = 1003)	819 (81,7)	70 (7,0)	72 (7,2)	36 (3,6)	*M* *=* 0,58 (*SD* *=* 1,42)
	Männer	406 (84,1)	32 (6,6)	27 (5,6)	18 (3,7)	–
	Frauen	413 (80,4)	38 (7,4)	45 (8,8)	18 (3,5)	χ^2^(3) = 4,114; *p* = 0,249
	18–29 Jahre	68 (58,1)	24 (20,5)	17 (14,5)	8 (6,8)	–
	30–39 Jahre	80 (77,7)	11 (10,7)	4 (3,9)	8 (7,8)	–
	40–49 Jahre	151 (79,1)	14 (7,3)	19 (9,9)	7 (3,7)	–
	50–59 Jahre	164 (86,3)	7 (3,7)	14 (7,4)	5 (2,6)	–
	60–69 Jahre	132 (91,7)	5 (3,5)	3 (2,1)	4 (2,8)	–
	70+ Jahre	224 (88,9)	9 (3,6)	15 (6,0)	4 (1,6)	χ^2^(15) = 84,253; *p* *<* 0,001
2020	Gesamt (*n* = 1050)	645 (61,4)	126 (12,0)	219 (20,9)	60 (5,7)	*M* *=* 1,25 (*SD* *=* 1,82)
	Männer	330 (64,1)	59 (11,5)	107 (20,8)	19 (3,7)	–
	Frauen	315 (58,9)	67 (12,5)	112 (20,9)	41 (7,7)	χ^2^(3) = 8,660; *p* *=* 0,034
	18–29 Jahre	67 (38,3)	38 (21,7)	55 (31,4)	15 (8,6)	–
	30–39 Jahre	85 (52,5)	29 (17,9)	39 (24,1)	9 (5,6)	–
	40–49 Jahre	104 (66,2)	16 (10,2)	33 (21,0)	4 (2,5)	–
	50–59 Jahre	143 (70,4)	16 (7,9)	32 (15,8)	12 (5,9)	–
	60–69 Jahre	101 (65,2)	15 (9,7)	29 (18,7)	10 (6,5)	–
	70+ Jahre	145 (73,2)	12 (6,1)	31 (15,7)	10 (5,1)	χ^2^(15) = 74,967; *p* *<* 0,001

*M* Mittelwert, *SD* Standardabweichung, *χ*^*2*^ Test auf Häufigkeitsunterschiede, *p* Signifikanzwert

**Abb. 1:**
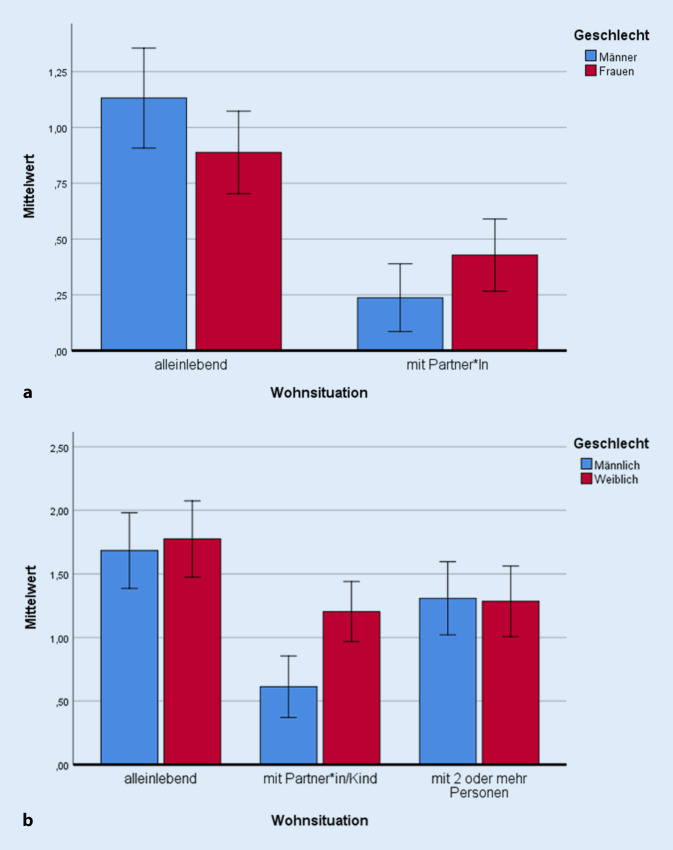
Interaktion aus Wohnsituation und Einsamkeit vor und während der Coronapandemie: **a** Gefühlte Einsamkeit gemessen im Jahr 2019, **b** gefühlte Einsamkeit gemessen im Jahr 2020 (Fehlerbalken = ±2 Standardabweichungseinheiten)

*Vor der Coronapandemie* gab es signifikante Unterschiede in der Wahrnehmung der Einsamkeit zwischen alleinlebenden Befragten (diese fühlten sich einsamer mit *M*_allein lebend_ = 0,987; *SD*_allein lebend_ = 1,766) und denjenigen in Partnerschaft/Wohngemeinschaft (fühlten sich weniger einsam mit *M*_gemeinsam lebend_ = 0,326; *SD*_gemeinsam lebend_ =1,089; *F*_Wohnsituation_[1; 993] = 54,922; *p* *<* 0,001; η^2^ = 0,052). Auch wenn Frauen und Männer sich nicht signifikant in ihrer Einsamkeit unterschieden (*F*_Geschlecht_[1; 993] = 0,084; *p* *=* 0,772; η^2^ = 0,001), war der Interaktionseffekt (Geschlecht*Wohnsituation) statistisch bedeutsam (*F*[3; 993] = 5,648; *p* *=* 0,018; η^2^ = 0,006). Wie in Abb. 1 dargestellt, fühlten sich vor der Coronakrise alleinlebende Männer einsamer als alleinlebende Frauen; in Partnerschaften berichteten Frauen häufiger einsam zu sein.

*Während der Coronapandemie* gab es ebenfalls signifikante Unterschiede in der Wahrnehmung der Einsamkeit zwischen alleinlebenden Menschen (fühlten sich häufiger einsam mit *M*_allein lebend_ = 1,729; *SD*_allein lebend_ = 2,031) und denjenigen, die mit einer Person zusammen lebten (weniger häufig einsam mit *M*_mit1 Person lebend_ = 0,926;* SD*_gemeinsam lebend_ = 1,648) sowie denjenigen, die mit mehr als einer Person zusammen lebten (*M*_mit 2 Personen lebend_ = 1,296; *SD*_wohnt mit2_ _Personen_ = 1,755; Abb. 1; *F*_Wohnsituation_[2;1044] = 18,584; *p* *<* 0,001; η^2^ = 0,034). Frauen fühlten sich generell signifikant einsamer als Männer (*F*_Geschlecht_[1;1044] = 3,829; *p* *=* 0,051; η^2^ = 0,004). Der Interaktionseffekt für Geschlecht und Wohnform war nur dann statistisch bedeutsam, wenn drei Beziehungsgruppen differenziert wurden (*F*_Geschlecht * Wohnsituation_[5;1044] = 3,240; *p* *=* 0,040; η^2^ = 0,006). Frauen und Alleinlebende berichteten generell höhere Einsamkeit (Abb. 1).

Für eine Übersicht der Korrelationen zwischen Einsamkeit bzw. Zunahme von Einsamkeit und Alter, Geschlecht, Wohnsituation, Ängsten und Sorgen, Bewältigungsstrategien sowie gesundheitlichen Belastungen während der Coronapandemie s. Tab. 2. Hierfür wurden Bewältigungsstrategien mittels Faktorenanalyse zusammengefasst (s. Anhang).

**Tab. 2:** Korrelationsmatrix aller untersuchten Items, die im Jahr 2020 erhoben wurden, mit Pearsons-Korrelationen (und *p*-Wert in Klammern) sowie Mittelwert (und Standardabweichung) in der ersten Spalte

Variable (Range)	*M (SD)*	1	2	3	4	5	6	7	8	9	10	11	12
1 Alter (18-90)	49,88 (17,33)	1	−0,058 (0,062)	−0,317 (<0,001)	−0,160 (<0,001)	−0,141 (<0,001)	−0,272 (<0,001)	−0,341 (<0,001)	0,274 (<0,001)	−0,052 (<0,001)	−0,305 (0,091)	−0,042 (0,174)	−0,252 (<0,001)
2 Geschlecht (Frauen = 1; Männer = 2; 1–2)	–	–	1	−0,016 (0,615)	−0,071 (0,021)	−0,069 (0,026)	−0,060 (0,054)	−0,131 (<0,001)	0,078 (0,011)	−0,120 (<0,001)	0,089 (0,004)	−0,109 (<0,001)	−0,051 (0,097)
3 Anzahl Personen im Haushalt (1–3)	2,03 (0,76)	–	–	1	−0,084 (0,006)	−0,061 (0,049)	0,140 (<0,001)	0,138 (<0,001)	−0,074 (0,017)	0,131 (<0,001)	0,134 (<0,001)	0,055 (0,073)	0,054 (0,080)
4 Einsamkeit (0–6)^a^	1,25 (1,82)	–	–	–	1	0,585 (<0,001)	0,524 (<0,001)	0,274 (<0,001)	−0,258 (<0,001)	−0,045 (0,143)	0,290 (<0,001)	0,223 (<0,001)	0,493 (<0,001)
5 Zunahme Einsamkeit (1–4)	2,00 (0,95)	–	–	–	–	1	0,476 (<0,001)	0,324 (<0,001)	−0,344 (<0,001)	0,027 (0,389)	0,260 (<0,001)	0,271 (<0,001)	0,432 (<0,001)
6 Ängste und Sorgen (15 Items; Cronbachs Alpha = 0,91; 1–4)	1,87 (0,58)	–	–	–	–	–	1	0,435 (<0,001)	−0,306 (<0,001)	0,159 (<0,001)	0,431 (<0,001)	0,485 (<0,001)	0,586 (<0,001)
7 Aktive Bewältigungsstrategien mittels Kommunikation (7 Items; Cronbachs Alpha = 0,79; 1–4)	2,19 (0,71)	–	–	–	–	–	–	1	−0,424 (<0,001)	0,327 (<0,001)	0,224 (<0,001)	0,286 (<0,001)	0,293 (<0,001)
8 Bewältigungsstrategie „nichts verändert“ (1 Item; 1–4)	2,41 (1,10)	–	–	–	–	–	–	–	1	−0,111 (<0,001)	−0,177 (<0,001)	−0,198 (<0,001)	−0,310 (<0,001)
9 Sinnfindung (3 Items; Cronbachs Alpha = 0,83; 1–4)	2,67 (0,75)	–	–	–	–	–	–	–	–	1	−0,079 (0,011)	0,331 (<0,001)	0,012 (0,700)
10 Eingeschränkte Beziehungsqualität (2 Items; *r* = 0,49; 1–4)	1,61 (0,72)	–	–	–	–	–	–	–	–	–	1	0,154 (<0,001)	0,291 (<0,001)
11 Sorgen um Lieben/Freund*innen (1 Item; 1–4)	2,46 (0,95)	–	–	–	–	–	–	–	–	–	–	1	0,262 (<0,001)
12 Gesundheitliche Belastungen (1 Item; 1–4)	1,81 (0,80)	–	–	–	–	–	–	–	–	–	–	–	1

^a^Die Einsamkeitsskala hat vier Ausprägungen: 0 (selten/nie), 1,5 (einmal die Woche), 3,5 (mehrfach pro Woche) und 6 (täglich). Alle anderen Variablen haben bei einer Spannbreite von 1–4 genau vier Ausprägungen

### Veränderung der Einsamkeit während der Coronapandemie (Fragestellung 2)

Im Zusammenhang mit der Coronapandemie berichteten 30,8 % der Befragten, dass sie sich *einsamer als vor den Einschränkungen fühlten*. Diejenigen, die sich einsamer als vor den Einschränkungen fühlten, berichteten auch generell während der Coronapandemie an mehr Tagen pro Woche einsam zu sein als Personen, die nicht angaben, seit der Coronapandemie einsamer zu sein (Tab. 2). Bei denjenigen, die sich während der Coronapandemie einsamer fühlten als noch vor den Einschränkungen, war der Anteil jüngerer Befragter größer als bei Annahme einer Unabhängigkeit erwartet wurde (χ^2^(15) = 46,013; *p* *<* 0,001). Unterschiede zwischen Frauen und Männern waren nicht signifikant (χ^2^(3) = 5,340; *p* *=* 0,149).

### Gesundheitliche Belastungen während der Coronapandemie (Forschungsfrage 3)

Gesundheitliche Belastungen im Zusammenhang mit den Einschränkungen erlebten 40,6 % der Befragten gar nicht. Weitere 40,6 % gaben an, dass es ihnen manchmal nicht gut ging. Weitere 16,2 % berichteten, dass sie relativ stark und zunehmend belastet waren. 2,6 % erklärten, dass sie sehr stark belastet waren und es ihnen sehr schlecht ging. Alle oben berücksichtigten Prädiktoren der Einsamkeit und des Einsamkeitsanstiegs inkl. dieser beiden Variablen (Tab. 2) wurden berücksichtigt, um Assoziationen mit dem Gefühl der gesundheitlichen Belastung (d. h. abhängige Variable) in einer linearen Regressionsanalyse zu untersuchen (Tab. 3). Alle berücksichtigten Variablen klärten R^2^ = 0,417 und adj. R^2^ = 0,410 der Varianz auf. Gesundheitliche Belastungen waren positiv mit berichteter Einsamkeitshäufigkeit (β = 0,190, *p* < 0,001) und einer wahrgenommenen Zunahme der Einsamkeit seit Beginn der Pandemie (β = 0,088, *p* = 0,003) assoziiert. Zudem gab es signifikante Zusammenhänge zwischen gesundheitlichen Belastungen und Ängsten (β = 0,412, *p* < 0,001). Dahingegen waren zunehmendes Alter (β = −0,084, *p* = 0,003), Sinnfindung (β = −0,057, *p* = 0,035) und die Angabe, dass nichts verändert wurde, um die eingeschränkten persönlichen sozialen Kontakte auszugleichen (β = −0,096, *p* < 0,001), negativ mit gesundheitlichen Einschränkungen assoziiert. Geschlecht und Anzahl von Mitbewohner*innen sowie aktive Bewältigungsstrategien, eingeschränkte Beziehungsqualität und Sorgen um Lieben/Freund*innen klärten bei gleichzeitiger Kontrolle aller anderen Variablen keinen signifikanten Anteil der Varianz auf.

**Tab. 3:** Ergebnisse der linearen Regressionsanalyse zur Vorhersage der gesundheitlichen Belastungen während der Coronapandemie

	B	SE	Beta	t	*p*
Konstante	1,115	0,176	–	6,322	<0,001
1 Alter	−0,004	0,001	−0,084	−3,020	0,003
2 Geschlecht	−0,021	0,039	−0,013	−0,547	0,584
3 Anzahl Personen im Haushalt	−0,006	0,027	−0,006	−0,2319	0,827
4 Einsamkeit	0,083	0,014	0,190	5,964	<0,001
5 Zunahme Einsamkeit	0,074	0,026	0,088	2,828	0,005
6 Ängste und Sorgen	0,565	0,048	0,412	11,817	<0,001
7 Aktive Bewältigungsstrategien	−0,018	0,034	−0,016	−0,529	0,597
8 Bewältigungsstrategie „nichts verändert“	−0,070	0,020	−0,096	−3,508	<0,001
9 Sinnfindung	−0,061	0,029	−0,057	−2,208	0,035
10 Eingeschränkte Beziehungsqualität	−0,006	0,030	−0,005	−0,181	0,856
11 Sorgen, um meine Lieben/Freund*innen	−0,003	0,024	−0,004	−0,125	0,900

*B* unstandardisierter Regressionskoeffizient. *SE* Standardfehler. *Beta* standardisierter Regressionskoeffizient. *p* Signifikanzwert, abhängige Variable: gesundheitliche Belastungen (1 Item)

## Diskussion und Ausblick

Hinsichtlich der ersten Fragestellung berichtete ein größerer Anteil der befragten Personen während der Coronapandemie von *häufigen Einsamkeitsgefühlen *im Vergleich zu befragten Personen vor der Pandemie. Von Einsamkeitsgefühlen berichteten insbesondere alleinlebende Personen. Dies deckt sich mit Befunden anderer Studien [1, 6, 15]. Vor der Coronapandemie unterschieden sich Frauen und Männer nur dann in ihrer wahrgenommenen Einsamkeit, wenn nach Partnerschaftsstatus differenziert wurde (Männer fühlten sich stärker einsam als Single; Frauen eher in einer Partnerschaft). Während der Coronapandemie fühlten sich Frauen und Alleinlebende unabhängig von einer Interaktion dieser zwei Faktoren häufiger einsam. Eine mögliche Erklärung ist, dass der Kontakt während der Coronapandemie wichtiger geworden ist, Frauen sich in Partnerschaften aufgrund höherer Erwartungen jedoch eher einsam fühlen als Männer [17]. Jüngere fühlten sich in beiden Befragungen häufiger einsam. Bezüglich der zweiten Fragestellung gab nahezu ein Drittel an, während der Coronapandemie eine *Veränderung von Einsamkeit* wahrzunehmen, wobei sich Altersunterschiede zeigten: Jüngere berichteten eher, dass sie im Zusammenhang mit der Coronapandemie einsamer geworden seien.

Einsamkeit korrelierte mit der Anwendung von Bewältigungsstrategien, das heißt im Schnitt berichteten diejenigen, die sich häufiger einsam fühlten, jeweils über mehr *Bewältigungsstrategien inklusive Kommunikation*, also Kontaktversuche, um Beziehungen zu anderen Menschen aufrechtzuerhalten oder wiederherzustellen – ganz im Einklang mit bisherigen Befunden und theoretischen Überlegungen [2]. Da es sich bei dem vorliegenden Datensatz um querschnittliche, korrelative Daten handelt, kann dies nur als Zeichen gesehen werden, dass diejenigen, die mehr Einsamkeit wahrnehmen, auch stärker versuchen, auf andere Menschen zuzugehen. Keinesfalls kann jedoch der Schluss gezogen werden, dass diejenigen, die nichts verändern, sich deswegen weniger einsam fühlen. Denn wahrscheinlich ist es genau entgegengesetzt: Diejenigen, die keine Diskrepanzen zwischen tatsächlichen und gewünschten Beziehungen wahrnehmen bzw. eine ausreichende Qualität und Quantität der sozialen Interaktionen empfinden, sind auch diejenigen, die sich nicht bzw. weniger einsam fühlen. Auch bezüglich* Einsamkeit* und *Ängsten oder Sorgen *zeigten sich Korrelationen: Wer sich mehr sorgte und mehr Konflikte in sozialen Beziehungen wahrnahm, fühlte sich eher einsam.

Außerdem sollte in der vorliegenden Studie ermittelt werden, inwiefern in Deutschland lebende Personen *während der Coronapandemie* Beschwerden in der subjektiven Gesundheit berichteten. Dabei wurde deutlich, dass ca. 60 % der Deutschen über gesundheitliche Belastungen während der Pandemie berichtet haben, wovon ca. ein Drittel sogar relativ stark bis sehr stark darunter litt. Einsamkeit, jüngeres Alter und mehr Sorgen waren mit wahrgenommenen gesundheitlichen Belastungen assoziiert. Wer jedoch, wie viele der Befragten, in der Herausforderung einen Sinn finden konnte, wie das Zusammenwachsen mit der Familie oder Wertschätzung sozialer Kontakte, war auch weniger dazu geneigt, gesundheitliche Belastungen im Zusammenhang mit den Pandemie-bedingten Einschränkungen zu erleben.

Einschränkend muss zu dieser Studie angemerkt werden, dass das gewählte Einsamkeitsmaß keinem standardisierten Item entsprach, sondern 2019 aufgrund von Pilotierungen leicht angepasst wurde und dann gleichermaßen in 2020 erhoben wurde. Damit ist ein Vergleich mit anderen Studien nur eingeschränkt möglich. Falls zukünftige Studien die hier verwendete Skala verwenden wollen, dann könnten wiederum Validierungsstudien feststellen, welche Vorteile die hier verwendete Messmethodik mit sich bringt. Vorsicht ist des Weiteren bei dem Vergleich mit Studien geboten, welche ein indirektes Maß für Einsamkeit genutzt haben im Vergleich zu dem in dieser Studie genutzten direkten Maß für Einsamkeit. Dies liegt darin begründet, dass die unterschiedlichen Maße zu verschiedenen Interpretationen hinsichtlich Einsamkeitsprävalenz und assoziierter Faktoren führen [20]. Daher sollten zukünftige Studien die Erfassung der Veränderung der Einsamkeit psychometrisch untersuchen und optimieren: In Pilotierungen war die Formulierung als passend festgestellt worden, jedoch könnte es angemessener sein, nicht nur nach einer Zunahme der Einsamkeit, sondern auch nach einer Abnahme und einem Gleichbleiben zu fragen, um eine Beeinflussung der Befragten und eine verzerrte Antwort zu vermeiden. Darüber hinaus könnte eine optimierte Messung der Konstrukte wie Einsamkeit und gesundheitliche Belastung über verschiedene Fragen erfolgen und damit Messfehler minimieren [21]. Als weitere Limitation ist die Unabhängigkeit der zwei untersuchten Stichproben zu nennen, aufgrund derer keine Längsschnittergebnisse berichtet und somit Aussagen zu Veränderungen der Einsamkeit nur eingeschränkt getroffen werden können. Als Stärken dieser Studie lassen sich jedoch die Größe und Repräsentativität der zwei Stichproben festhalten, ebenso wie der Einschluss psychologischer Konstrukte in die Untersuchung einer Veränderung der wahrgenommenen Einsamkeit sowie der erlebten gesundheitlichen Belastungen. Mithilfe der Ergebnisse können relevante Aussagen über die erlebten Einschränkungen, assoziierten Faktoren und angewandten Bewältigungsstrategien getroffen werden und Handlungsempfehlungen abgeleitet werden:

Die meisten Menschen haben die Einschränkungen – bis Juni 2020 – gut überstanden, was v. a. durch die Nutzung von Bewältigungs- und Kommunikationsstrategien geschehen konnte. Das Umsteigen auf sichere digitale Kommunikation kann insbesondere in Zeiten von COVID-19 eine gute Möglichkeit sein, Verbundenheit und soziale Unterstützung aufrechtzuerhalten, was ein protektiver Faktor gegen Gesundheitseinschränkungen (sowohl psychisch als auch physisch) sein kann [22–24]. Eine wichtige Bewältigungsstrategie ist die Sinnfindung: Insbesondere nach Phasen, die durch erhöhte Anforderungen und Stress gekennzeichnet sind, nehmen viele Menschen auch persönliches Wachstum und positive Konsequenzen wahr [13].

Dennoch zeigte die Coronapandemie auch deutliche Zusammenhänge mit verschiedenen Gedanken und Gefühlen. Es wurde eine hohe Risikowahrnehmung in Form einer subjektiv eingeschätzten Zugehörigkeit zur Risikogruppe bei knapp 50 % der untersuchten, repräsentativen Stichprobe beobachtet. Dies ist im Vergleich zum als eher gering eingeschätzten wahrgenommenen Risiko bezüglich der Influenza sehr hoch [1]. Insbesondere die Personen, die sich verstärkt Sorgen machten (v. a. Jüngere) litten unter Einsamkeit. *Jüngeres Alter* war auch in anderen Studien zur Coronapandemie ein Risikofaktor für höhere Einsamkeit [1, 6, 14]. Die erhöhte Vulnerabilität der Jüngeren kann dadurch erklärt werden, dass diese Gruppe möglicherweise eine geringere Resilienz gegenüber Stressoren [18] bzw. eine weniger effektive Emotionsregulation [5] aufweist. Zusätzlich zur Einsamkeit sind Distanzunterricht und Homeoffice/mobiles Arbeiten einschneidende Herausforderungen, was v. a. die *RHOL *betrifft. Im Alter zwischen 30 und 39 Jahren sind viele Personen durch Familie und Beruf stark eingebunden und die Arbeitszeit übersteigt die Freizeit (auch durch unbezahlte Arbeit; [25]). Insbesondere diese Altersgruppe und ihre Bedürfnisse müssen daher in den Maßnahmen des Infektionsschutzes stärker berücksichtigt werden, um eine Zunahme von Einsamkeit und Morbidität zu vermeiden. Gleichzeitig sollten Menschen, die in solchen Ausnahmezeiten wie der Coronapandemie oder eines (Teil‑)Lockdowns ein Schicksal wie den Tod eines geliebten Mitmenschen oder den Verlust der Arbeit erleben, auch (weiterhin) unterstützt werden können: Unter diesen Beeinträchtigungen kann sich die Belastung potenzieren und das Leiden im Vergleich zu uneingeschränkten Zeiten verstärken, wenn nicht ausreichend soziale Unterstützung gegeben werden kann [4]. Entsprechend sollten hier langfristige Lösungen gefunden werden, bei denen beispielsweise die Digitalisierung einen entscheidenden Faktor übernehmen kann.

## Fazit für die Praxis

- Aufgrund des Zusammenhangs von Sorgen, Einsamkeit und subjektivem Gesundheitsstatus sollten zukünftige Maßnahmen zur Prävention und Gesundheitsförderung die Rolle von spezifischen Ängsten in der Coronapandemie berücksichtigen.
- In der vorliegenden Studie wurde Einsamkeit als zentraler Faktor betrachtet, da Einsamkeit negativ mit der psychischen sowie körperlichen Gesundheit zusammenhängen kann.
- Diejenigen, die sich im Zusammenhang mit den Einschränkungen deutlich be- bzw. überlastet fühlen, sollten im Blickfeld behalten und bei der Bewältigung der Herausforderungen unterstützt werden.
- Kompetenzaufbau, Ressourcenstärkung, sichere Kommunikation und gezielte Hilfe bei Krisen stellen dabei mögliche, nachhaltige Ansätze dar.

## Anhang

### 1. Faktorenanalyse mit aktiven Bewältigungsstrategien mittels Kommunikation und der Bewältigungsstrategie „nichts verändert“

**Tab. 4:** Deskriptive Statistiken

	*M*	*SD*	*n*
Ich habe mehr telefoniert	2,52	1,048	1050
Ich war häufiger in privaten Videocalls	1,86	1,059	1050
Ich war verstärkt in den sozialen Netzwerken (z. B. Facebook, Instagram) unterwegs	2,14	1,080	1050
Ich habe mehr Text‑/Sprachnachrichten versendet	2,36	1,091	1050
Ich habe mehr Fotos gepostet/geschickt	1,92	1,033	1050
Ich habe häufiger aus dem Fenster geschaut	2,22	1,079	1050
Ich habe mehr Serien geschaut	2,30	1,096	1050
Ich habe nichts verändert	2,41	1,099	1050

**Tab. 5:** Korrelationsmatrix

	Ich habe mehr telefoniert	Ich war häufiger in privaten Videocalls	Ich war verstärkt in den sozialen Netzwerken (z. B. Facebook, Instagram) unterwegs	Ich habe mehr Text‑/Sprachnachrichten versendet	Ich habe mehr Fotos gepostet/geschickt	Ich habe häufiger aus dem Fenster geschaut	Ich habe mehr Serien geschaut	Ich habe nichts verändert
Ich habe mehr telefoniert	1,000	–	–	–	–	–	–	–
Ich war häufiger in privaten Videocalls	0,328	1,000	–	–	–	–	–	–
Ich war verstärkt in den sozialen Netzwerken (z. B. Facebook, Instagram) unterwegs	0,221	0,383	1,000	–	–	–	–	–
Ich habe mehr Text‑/Sprachnachrichten versendet	0,357	0,411	0,510	1,000	–	–	–	–
Ich habe mehr Fotos gepostet/geschickt	0,296	0,375	0,477	0,586	1,000	–	–	–
Ich habe häufiger aus dem Fenster geschaut	0,206	0,222	0,330	0,409	0,379	1,000	–	–
Ich habe mehr Serien geschaut	0,180	0,219	0,370	0,368	0,281	0,409	1,000	–
Ich habe nichts verändert	−0,178	−0,228	−0,312	−0,340	−0,229	−0,320	−0,358	1,000

Alle Korrelationen *p* < 0,001

**Tab. 6:** Erklärte Gesamvarianz

Komponente	Anfängliche Eigenwerte			Summen von quadrierten Faktorladungen für Extraktion			Rotierte Summen der quadrierten Ladungen		
	Gesamt	% der Varianz	Kumulierte %	Gesamt	% der Varianz	Kumulierte %	Gesamt	% der Varianz	Kumulierte %
1	3,373	42,157	42,157	3,373	42,157	42,157	2,285	28,568	28,568
2	1,025	12,810	54,967	1,025	12,810	54,967	2,112	26,399	54,967
3	0,794	9,930	64,898	–	–	–	–	–	–
4	0,723	9,037	73,934	–	–	–	–	–	–
5	0,622	7,772	81,706	–	–	–	–	–	–
6	0,586	7,330	89,036	–	–	–	–	–	–
7	0,483	6,043	95,079	–	–	–	–	–	–
8	0,394	4,921	100,000	–	–	–	–	–	–

Extraktionsmethode: Hauptkomponentenanalyse. Die Komponenten stehen für die einzelnen Bewältigungsstrategien

**Tab. 7:** Rotierte Komponentenmatrix^a^

	Komponente	
	1	2
Ich habe mehr telefoniert	0,692	–
Ich war häufiger in privaten Videocalls	0,729	–
Ich war verstärkt in den sozialen Netzwerken (z. B. Facebook, Instagram) unterwegs	0,535	0,478
Ich habe mehr Text‑/Sprachnachrichten versendet	0,670	0,449
Ich habe mehr Fotos gepostet/geschickt	0,683	–
Ich habe häufiger aus dem Fenster geschaut	–	0,693
Ich habe mehr Serien geschaut	–	0,771
Ich habe nichts verändert	–	−0,697

Extraktionsmethode: Hauptkomponentenanalyse

Rotationsmethode: Varimax mit Kaiser-Normalisierung

^a^Die Rotation ist in drei Iterationen konvergiert

### 2. Faktorenanalyse mit Items zu Sinnfindung, eingeschränkter Beziehungsqualität und Sorgen um die Lieben/Freund*innen

**Tab. 8:** Deskriptive Statistiken

	*M*	*SD*	*n*
Wir sind enger zusammengewachsen, geben uns Halt	2,67	0,871	1050
Ich habe meine Familie/mein soziales Umfeld mehr zu schätzen gelernt	2,77	0,868	1050
Soziale Konflikte durch zu viel Nähe haben zugenommen (z. B. Streit, rauer Ton, Unruhe)	1,66	0,875	1050
Wir reden mehr miteinander	2,58	0,870	1050
Wir haben uns voneinander distanziert	1,56	0,798	1050
Ich habe mir viele Sorgen, um meine Lieben/meine Freund*innen gemacht	2,46	0,953	1050

**Tab. 9:** Korrelationsmatrix

	Wir sind enger zusammengewachsen, geben uns Halt	Ich habe meine Familie/mein soziales Umfeld mehr zu schätzen gelernt	Soziale Konflikte durch zu viel Nähe haben zugenommen (z. B. Streit, rauer Ton, Unruhe)	Wir reden mehr miteinander	Wir haben uns voneinander distanziert	Ich habe mir viele Sorgen, um meine Lieben/meine Freund*innen gemacht
Wir sind enger zusammengewachsen, geben uns Halt	1,000	–	–	–	–	–
Ich habe meine Familie/mein soziales Umfeld mehr zu schätzen gelernt	0,690**	1,000	–	–	–	–
Soziale Konflikte durch zu viel Nähe haben zugenommen (z. B. Streit, rauer Ton, Unruhe)	−0,030	0,056*	1,000	–	–	–
Wir reden mehr miteinander	0,595**	0,567**	0,069*	1,000	–	–
Wir haben uns voneinander distanziert	−0,217**	−0,127**	0,493**	−0,130**	1,000	–
Ich habe mir viele Sorgen, um meine Lieben/meine Freund*innen gemacht	0,268**	0,334**	0,178**	0,255**	0,085**	1,000

**p < 0,01

**Tab. 10:** Erklärte Gesamtvarianz

Komponente	Anfängliche Eigenwerte			Summen von quadrierten Faktorladungen für Extraktion			Rotierte Summen der quadrierten Ladungen		
	Gesamt	% der Varianz	Kumulierte %	Gesamt	% der Varianz	Kumulierte %	Gesamt	% der Varianz	Kumulierte %
1	2,449	40,813	40,813	2,449	40,813	40,813	2,262	37,699	37,699
2	1,565	26,075	66,888	1,565	26,075	66,888	1,506	25,108	62,807
3	0,762	12,698	79,586	0,762	12,698	79,586	1,007	16,779	79,586
4	0,481	8,023	87,609	–	–	–	–	–	–
5	0,444	7,408	95,017	–	–	–	–	–	–
6	0,299	4,983	100,000	–	–	–	–	–	–

Extraktionsmethode: Hauptkomponentenanalyse. Die Komponenten stehen für die Items zu Sinnfindung, eingeschränkter Beziehungsqualität und Sorgen um die Lieben/Freund*innen

**Tab. 11:** Rotierte Komponentenmatrix^a^

	Komponente		
	1	2	3
Wir sind enger zusammengewachsen, geben uns Halt	0,870	–	–
Ich habe meine Familie/mein soziales Umfeld mehr zu schätzen gelernt	0,846	–	–
Soziale Konflikte durch zu viel Nähe haben zugenommen (z. B. Streit, rauer Ton, Unruhe)	–	0,874	–
Wir reden mehr miteinander	0,839	–	–
Wir haben uns voneinander distanziert	–	0,846	–
Ich habe mir viele Sorgen, um meine Lieben/meine Freund*innen gemacht	–	–	0,971

Extraktionsmethode: Hauptkomponentenanalyse

Rotationsmethode: Varimax mit Kaiser-Normalisierung

^a^Die Rotation ist in vier Iterationen konvergiert

## Footnotes

1 http://www.drktg.de/mz/pdf/downloads/CES-D.pdf.
